# Bile acids induce necrosis in pancreatic stellate cells dependent on calcium entry and sodium‐driven bile uptake

**DOI:** 10.1113/JP272774

**Published:** 2016-08-08

**Authors:** Pawel E. Ferdek, Monika A. Jakubowska, Julia V. Gerasimenko, Oleg V. Gerasimenko, Ole H. Petersen

**Affiliations:** ^1^ Medical Research Council GroupCardiff School of Biosciences, Cardiff UniversityCardiffCF10 3AXWalesUK; ^2^ Systems Immunity Research InstituteCardiff UniversityCardiffCF14 4XNWalesUK

**Keywords:** bile, calcium, inflammation, necrosis, pancreas, pancreatitis, stellate cells

## Abstract

**Key points:**

- Acute biliary pancreatitis is a sudden and severe condition initiated by bile reflux into the pancreas.
- Bile acids are known to induce Ca^2+^ signals and necrosis in isolated pancreatic acinar cells but the effects of bile acids on stellate cells are unexplored.
- Here we show that cholate and taurocholate elicit more dramatic Ca^2+^ signals and necrosis in stellate cells compared to the adjacent acinar cells in pancreatic lobules; whereas taurolithocholic acid 3‐sulfate primarily affects acinar cells.
- Ca^2+^ signals and necrosis are strongly dependent on extracellular Ca^2+^ as well as Na^+^; and Na^+^‐dependent transport plays an important role in the overall bile acid uptake in pancreatic stellate cells.
- Bile acid‐mediated pancreatic damage can be further escalated by bradykinin‐induced signals in stellate cells and thus killing of stellate cells by bile acids might have important implications in acute biliary pancreatitis.

**Abstract:**

Acute biliary pancreatitis, caused by bile reflux into the pancreas, is a serious condition characterised by premature activation of digestive enzymes within acinar cells, followed by necrosis and inflammation. Bile acids are known to induce pathological Ca^2+^ signals and necrosis in acinar cells. However, bile acid‐elicited signalling events in stellate cells remain unexplored. This is the first study to demonstrate the pathophysiological effects of bile acids on stellate cells in two experimental models: *ex vivo* (mouse pancreatic lobules) and *in vitro* (human cells). Sodium cholate and taurocholate induced cytosolic Ca^2+^ elevations in stellate cells, larger than those elicited simultaneously in the neighbouring acinar cells. In contrast, taurolithocholic acid 3‐sulfate (TLC‐S), known to induce Ca^2+^ oscillations in acinar cells, had only minor effects on stellate cells in lobules. The dependence of the Ca^2+^ signals on extracellular Na^+^ and the presence of sodium–taurocholate cotransporting polypeptide (NTCP) indicate a Na^+^‐dependent bile acid uptake mechanism in stellate cells. Bile acid treatment caused necrosis predominantly in stellate cells, which was abolished by removal of extracellular Ca^2+^ and significantly reduced in the absence of Na^+^, showing that bile‐dependent cell death was a downstream event of Ca^2+^ signals. Finally, combined application of TLC‐S and the inflammatory mediator bradykinin caused more extensive necrosis in both stellate and acinar cells than TLC‐S alone. Our findings shed new light on the mechanism by which bile acids promote pancreatic pathology. This involves not only signalling in acinar cells but also in stellate cells.

## Key points

- Acute biliary pancreatitis is a sudden and severe condition initiated by bile reflux into the pancreas.
- Bile acids are known to induce Ca^2+^ signals and necrosis in isolated pancreatic acinar cells but the effects of bile acids on stellate cells are unexplored.
- Here we show that cholate and taurocholate elicit more dramatic Ca^2+^ signals and necrosis in stellate cells compared to the adjacent acinar cells in pancreatic lobules; whereas taurolithocholic acid 3‐sulfate primarily affects acinar cells.
- Ca^2+^ signals and necrosis are strongly dependent on extracellular Ca^2+^ as well as Na^+^; and Na^+^‐dependent transport plays an important role in the overall bile acid uptake in pancreatic stellate cells.
- Bile acid‐mediated pancreatic damage can be further escalated by bradykinin‐induced signals in stellate cells and thus killing of stellate cells by bile acids might have important implications in acute biliary pancreatitis.

**Abbreviations**
α‐SMA: α‐smooth muscle actin
ACh: acetylcholine
AM: acetoxymethyl
AP: acute pancreatitis
ASBT: apical sodium‐dependent bile acid transporter
ATP: adenosine triphosphate
BK: bradykinin
BDKRB1: bradykinin receptor type 1
BDKRB2: bradykinin receptor type 2
CCK: cholecystokinin
DAPI: 4ʹ,6‐diamidino‐2‐phenylindole
ECM: extracellular matrix
ER: endoplasmic reticulum
hPSC: human pancreatic stellate cell
IHF: immunohistofluorescence
IP_3_R: inositol 1,4,5‐trisphosphate receptor
NaChol: sodium cholate
NMDG: *N*‐methyl‐d‐glucamine
NTCP: sodium–taurocholate cotransporting polypeptide
PAC: pancreatic acinar cell
PI: propidium iodide
PSC: pancreatic stellate cell
RyR: ryanodine receptor
SOAT: sodium‐dependent organic anion transporter
SOCE: store‐operated calcium entry
TBS‐T: Tris‐buffered saline with 0.1% Tween 20
TC: sodium taurocholate
TLC‐S: taurolithocholic acid 3‐sulfate

## Introduction

Acute pancreatitis (AP) is a potentially severe disease with an overall mortality up to 6% (de Beaux *et al*. 1995; Gislason *et al*. 2004), and increases the risk of developing pancreatic cancer (Munigala *et al*. 2014). Even though we have known about AP for over 350 years, its pathogenesis is still debated and there is no specific treatment (Pannala *et al*. 2009; Takacs *et al*. 2013). Bile acids and gallstones have long been implicated in the pathogenesis of AP (Opie, 1901). A transient obstruction of the hepatopancreatic ampulla by gallstones can cause reflux of the bile from the gallbladder into the pancreatic duct (Armstrong & Taylor, 1986; Neoptolemos, 1989). Retrograde infusion of bile acids into the pancreatic duct has been shown to induce pancreatitis and is a well‐established model of the disease in rodents (Perides *et al*. 2010).

For many decades we have accumulated substantial knowledge about pancreatic acinar cells (PACs), especially in the context of pancreatic enzyme secretion, which, in physiological conditions, is induced by acetylcholine (ACh) or cholecystokinin (CCK) and regulated by intracellular Ca^2+^ signals (Case, 1973; Case & Clausen, 1973; Matthews *et al*. 1973; Petersen & Ueda, 1976; Petersen & Tepikin, 2008). PACs are polarised, with distinct apical and basolateral poles. The acinar nucleus and most of the endoplasmic reticulum (ER) is located in the basolateral region, whereas the secretory granules are confined to the significantly smaller apical pole, surrounded by a mitochondrial belt (Tinel *et al*. 1999). Ca^2+^ signals induced by low physiological doses of agonists are predominantly restricted to the apical pole, or become only transiently global, and are sufficient for stimulation of enzyme secretion (Maruyama *et al*. 1993; Thorn *et al*. 1993; Gerasimenko *et al*. 1996). In contrast, bile acids, such as taurolithocholic acid 3‐sulfate (TLC‐S), were demonstrated to cause large abnormal Ca^2+^ signals in PACs via a mechanism that involves depletion of intracellular Ca^2+^ stores and activation of Ca^2+^ entry (Voronina *et al*. 2002; Lau *et al*. 2005) as well as depolarisation of mitochondria (Voronina *et al*. 2005). Sustained Ca^2+^ elevations in acinar cells have been linked to premature intracellular enzyme activation, vacuolisation and cell necrosis (Kruger *et al*. 2000; Raraty *et al*. 2000). Those processes are the hallmark of the initial stages of acute pancreatitis.

Although cells relevant to exocrine and endocrine functions of the pancreas have been extensively studied and well described in the literature (Hegyi & Petersen, 2013; Petersen & Verkhratsky, 2016), a type of pancreatic auxiliary cells was overlooked until recently. Initially observed in 1982 (Watari *et al*. 1982) as vitamin A storing cells, then identified, characterised and isolated for the first time in 1998 (Apte *et al*. 1998; Bachem *et al*. 1998), pancreatic stellate cells (PSCs) are currently attracting interest predominantly due to their well‐documented role in pancreatic fibrosis (Apte *et al*. 2012, 2015). In the normal pancreas, PSCs exhibit a so‐called quiescent state and have limited capacity to migrate and proliferate. Importantly, they maintain extracellular matrix (ECM) turnover via synthesis and secretion of ECM proteins as well as its degrading enzymes (Phillips *et al*. 2003). Activation of PSCs, predominantly induced by tissue damage, triggers a transition to a myofibroblast‐like phenotype, expression of markers such as α‐smooth muscle actin (α‐SMA) and an increase in proliferation, migration as well as synthesis and secretion of ECM proteins (Apte *et al*. 1998; Bachem *et al*. 1998). Persisting injury of the pancreas, as present in chronic pancreatitis or pancreatic adenocarcinoma, causes severe imbalance between ECM production and degradation leading to excessive deposition of ECM components and development of fibrosis (Haber *et al*. 1999; Casini *et al*. 2000; Neuschwander‐Tetri *et al*. 2000). Interestingly, pancreatic fibrosis is a typical complication of alcohol‐induced pancreatitis, but data show that it is much less common in acute biliary pancreatitis (Pareja *et al*. 2003; Bertilsson *et al*. 2015; Ahmed Ali *et al*. 2016).

Even though in recent years substantial advances in the PSC field have been made, Ca^2+^ signalling in these cells was investigated only in a very limited number of studies (Won *et al*. 2011; Gryshchenko *et al*. 2016 *a*) and detailed signalling events in PSCs during development of pancreatitis remain largely unexplored. Evidence shows that bile acids may act on other cell types in the pancreas, such as duct cells, by inducing pathological Ca^2+^ signals and affecting ductal secretion (Venglovecz *et al*. 2008; Maleth *et al*. 2011; Hegyi & Rakonczay, 2015). Therefore, it is impossible to gain a full understanding of the processes fuelling the disease without a detailed knowledge of the effects bile acids exert on different cell types of the pancreas. Here we demonstrate for the first time the adverse effects of natural components of the bile on pancreatic stellate cells, provide new insights into the mechanism of bile acid‐induced pathology and draw conclusions about their implications in acute biliary pancreatitis.

## Methods

### Ethical approval

All procedures involving animals were performed in accordance with the UK Home Office regulations. In this study, however, no experiments were done on live animals. C57BL/6J mice (male, 6–8 weeks old, 23 ± 3 g weight) were supplied by Charles River, maintained on a standard rodent chow diet with free access to water, and housed in the institutional animal unit (12 h light cycle). The mice were killed according to Schedule 1 of Animals (Scientific Procedures) Act 1986, dissected and the pancreatic tissue was removed for further experimental procedures. In order to reduce the number of animals used in the study, where applicable, some experiments were performed on cells cultured *in vitro*. The investigators understand the ethical principles under which *The Journal of Physiology* operates and state that this work complies with these principles.

### Reagents

The main reagents for cell isolation and microscopy include: Fluo‐4 AM, propidium iodide and Hoechst‐33342 (ThermoFisher Scientific, Paisley, UK); collagenase type V, inorganic salts and bile salts (all from Sigma‐Aldrich, Gillingham, UK): sodium cholate (NaChol), sodium taurocholate (TC) and taurolithocholic acid 3‐sulfate (TLC‐S). NaHepes buffer was prepared as follows (mm): NaCl 140, KCl 4.7, Hepes 10, MgCl_2_ 1, glucose 10; pH 7.3. NMDG‐Hepes was a modification of NaHepes, where NaCl was replaced by 140 mm *N*‐methyl‐d‐glucamine (NMDG^+^), pH 7.3.

### Isolation of pancreatic lobules and loading with Fluo‐4

Lobule preparation and most of the experimental work was carried out in NaHepes buffer. Unless otherwise stated, NaHepes was supplemented with 1 mm Ca^2+^. The pancreas was isolated from a C57BL/6J mouse, washed twice in NaHepes, injected with type V collagenase (31.25 CDU ml^−1^, in NaHepes) and subsequently incubated at 37°C for 5–6 min in the collagenase solution to allow for partial digestion of the tissue. After incubation the pancreas was broken down by pipetting, suspended in NaHepes, spun (1 min, 0.2 × *g*), resuspended in NaHepes and spun again. Finally, isolated pancreatic lobules were suspended in NaHepes and loaded with Fluo‐4 AM as described below.

### Cell culture

Human pancreatic stellate cells (hPSCs) and stellate cell complete medium (SteCM) were obtained from ScienCell, Carlsbad, CA, USA. hPSCs were cultured in SteCM in T25 flasks at 37°C, 5% CO_2_ and split once a week. A frozen stock of hPSC was prepared after the first passage and was used to revive the culture every 5–6 weeks.

### Cytosolic Ca^2+^ measurements

Isolated mouse pancreatic lobules were loaded with 10 μm Fluo‐4 AM (stock in DMSO, further dissolved in NaHepes) for 1 h at 30°C. After the incubation pancreatic lobules were resuspended in fresh NaHepes and used for experiments at room temperature in a flow chamber perfused with NaHepes‐based extracellular solution. hPSCs were plated on sterile round coverslips and loaded with 1 μm Fluo‐4 AM for 30 min at 37°C. After the incubation the glass coverslips with hPSCs were used for the flow chamber assembly. Ca^2+^ imaging was performed using Leica confocal microscope TCS SPE with a ×63 oil objective. Fluo‐4 AM was excited with a 488 nm laser at 1–3% power and emitted light was collected at 500–600 nm. Static cell images were taken at 512 × 512 pixel resolution and series of images were recorded at 256 × 256, two consecutive frames were averaged, and time resolution was 1 image per 2 s. Fluorescence signals were plotted as *F*/*F*_0_, where *F*_0_ was an averaged signal from the first ten baseline images. For very long experiments linear correction of focus drift was applied.

### Cell death assay

For experiments with 30 min incubation pancreatic lobules were isolated and loaded with Fluo‐4 AM. Then the cells were perfused in a flow chamber with NaHepes‐ or NMDG‐Hepes‐based solution containing bile acid salts (with or without Ca^2+^). Ten minutes before the end of the incubation, perfusion was stopped and propidium iodide (PI) was added (2 μg ml^−1^).

In experiments involving long incubations (2 h) pancreatic lobules were kept in NaHepes or NMDG‐Hepes in the presence of bile acid. After the first hour, 10 μm Fluo‐4 was added and the incubation continued for another hour. 15 min before the end of the incubation PI (1 μg ml^−1^) and Hoechst‐33342 (5 μg ml^−1^) were added. Cells were visualised with Leica confocal microscope TCS SPE. Fluo‐4 fluorescence allowed for detection of PSCs in pancreatic lobules, PI specifically stained necrotic cells (excitation 535 nm, collected emission 585–705 nm) and Hoechst marked all nuclei (excitation 405, collected emission 420–480 nm) making possible calculation of the total number of cells. Pancreatic lobules were imaged by collecting multiple pictures along *z* axis, 5 μm apart. Then the pictures were merged yielding a maximum projection image, where live and necrotic cells were counted. Five to ten series were collected per sample.

hPSCs were plated on 35 mm glass bottom microwell dishes (MatTek, Ashland, MA, USA) and grown for 24 h in SteCM at 37°C, 5% CO_2_. Then the medium was replaced by NaHepes or NMDG‐Hepes containing 0.1 or 1 mm of NaChol or TC; and hPSCs were incubated for 2 h at 37°C. Fifteen minutes before the end of the incubation PI (1 μg ml^−1^) and Hoechst‐33342 (5 μg ml^−1^) were added. PI stained necrotic cells and Hoechst stained nuclei. Multiple pictures (15–20) per treatment group were taken; live, apoptotic and necrotic cells were counted in each treatment group.

### RT‐PCR and conventional PCR

Total RNA was extracted from hPSCs using the PureLink RNA Mini Kit (ThermoFisher Scientific). Human hepatocyte cDNA was obtained from ScienCell. Reverse transcription was performed with the GoScript Reverse Transcription System (Promega, Southampton, UK). cDNA was amplified using GoTaq G2 DNA Polymerase (Promega) and specific gene primer pairs (given in forward/reverse order) for *slc10A1* (CGT CCT CAA ATC CAA ACG GC/ACT TCA GGT GGA AAG GCC AC), human *desmin* (GAT CCA GTC CTA CAC CTG CG/CTC GGA AGT TGA GGG CAG AG), *bdkrb1* (TGG GAC CAC AGG TCA CTG/CCA GGT TGG CCA GGT AGA TT), *bdkrb2* (CTG TTC GTG AGG ACT CCG TG/AGG TAG ATC TCT GCC ACC GT), *slco4A1* (ATC TAC ACG GAA ATG GGC CG/ACA TGC CGG TGA TGA GAG TG), *slco1B3* (TGG CTT GGT TTC CTT GTG TC/CCA GTT GCA ACC GTA GGA AT) and *α‐sma* (TTC CAG CCA TCC TTC ATC GG / CCC GGC TTC ATC GTA TTC CT). The PCR reaction conditions were as follows: 5 min 95°C; then 40 cycles of 30 s 95°C, 1 min 56°C, 1 min 72°C; and finally 5 min 72°C. The PCR products were resolved on 1% agarose gel with Gel Red Nucleic Acid Gel Stain (Biotium, Hayward, CA, USA).

### Protein isolation and immunoblotting

Unless otherwise stated all reagents for immunoblotting mentioned below were obtained from ThermoFisher Scientific. Total protein was isolated from hPSCs, mouse pancreas, liver, kidney, spleen, lungs and suspended in RIPA buffer (Sigma‐Aldrich). Human liver tissue lysate was obtained from Novus Biologicals, Littleton, CO, USA. Protein concentration was determined using DC Protein Assay (Bio‐Rad, Hertfordshire, UK). For each sample the volume containing 75 μg of protein was brought to 26 μl by addition of double‐distilled water (ddH_2_O); then 4 μl of sample reducing agent and 10 μl 4 × NuPage LDS sample buffer were added. Samples were heated at 95°C for 10 min and then spun (5 min, 8000 × *g*). Proteins were separated by SDS‐PAGE (Bis‐Tris protein gel 4–12%, NuPage Mops SDS Running Buffer, 55 min, 200 V), then transferred on nitrocellulose membrane 0.45 μm (in NuPage Transfer Buffer, 25 V, 2 h), followed by 1 h incubation in 5% milk in Tris‐buffered saline with 0.1% Tween 20 (TBS‐T, Sigma‐Aldrich). Primary antibody anti‐NTCP (Abcam, Cambridge, UK) was prepared in a 1:2000 dilution in TBS‐T with 1% milk and incubated for 1 h. Membranes were then washed (3 × 10 min in TBS‐T) and incubated for 1 h in a 1:5000 dilution of HRP‐conjugated goat anti‐rabbit IgG secondary antibody (Santa Cruz Biotechnology, Dallas, TX, USA) in TBS‐T with 1% milk. The membranes were washed (4 × 10 min in TBS‐T) and then visualised using ImmunoCruz Western Blotting Luminol Reagent (Santa Cruz Biotechnology) and ChemiDoc XRS+ Imaging System (Bio‐Rad).

### Immunohistofluorescence

Formalin‐fixed paraffin‐embedded tissue sections (4 μm) were heated in a dry oven (30 min, 65°C), and then deparaffinised in xylene (2 × 10 min). The sections were rehydrated by subsequent washes in ethanol solutions with increasing content of ddH_2_O: 2 × 100%, 95%, 70%, 50%, and finally in ddH_2_O; and then incubated in 50 mm NH_4_Cl in ddH_2_O (20 min). Antigen retrieval was performed in TAE buffer (pH 8.1) in an autoclave (20 min, 120°C) and then the sections were allowed to cool at room temperature (30 min) before permeabilisation in 0.4% Triton X‐100 in ddH_2_O (10 min). Then the sections were washed 3 × 5 min in 0.1% Tween 20 in ddH_2_O (washing solution). In order to quench autofluorescence the sections were incubated in 0.2% Sudan Black B for 20 min (Sun *et al*. 2011) and then washed in the washing solution (4 × 5 min). Blocking of non‐specific binding sites was performed by 1 h incubation in the blocking buffer (1% bovine serum albumin in PSB with 0.1% Tween 20). Then the sections were incubated with the primary antibody (0.5 μg ml^−1^ or 1:200 dilution in the blocking buffer), initially for 1 h at room temperature, followed by an overnight incubation at 4°C inside a humid chamber. Negative controls were incubated in the blocking solution without antibody. The following day the sections were washed in the washing solution (4 × 5 min), and incubated for 1 h at room temperature with the appropriate secondary antibody (4 μg ml^−1^ or 1:500 in the blocking buffer). After the incubation the sections were washed in the washing solution (4 × 5 min), embedded in ProLong Diamond Antifade Mountant with DAPI (ThermoFisher Scientific) and imaged immediately using Leica sp5 confocal microscope. The slides were then stored at 4°C.

Primary antibodies: rabbit polyclonal anti‐NTCP antibody (a kind gift of Dr M. Ananthanarayanan, Yale University) (Ananthanarayanan *et al*. 1994); mouse monoclonal anti‐BDKRB2 antibody (sc‐136216, Santa Cruz Biotechnology). Secondary antibody: AlexaFluor 488 goat anti‐rabbit and AlexaFluor 635 goat anti‐mouse (ThermoFisher Scientific).

### Statistical analysis

For cell death assays, three to six independent experiments were performed for each treatment group on cells isolated from different animals; average values and standard errors of the mean were calculated and results presented as bar charts. Statistical analysis was performed using the Student's *t* test. For quantitative analysis of Ca^2+^ responses, areas under individual traces (over baseline) recorded between 200 and 2000 s were calculated and then averaged and presented as bar charts with standard errors. The Student's *t* test was applied for statistical comparison. The significance threshold was set at 0.05. Where applicable, *N* indicates the number of individual experiments/cell isolations, whereas *n* indicates individual cells.

## Results

### A brief characterisation of PSCs

**Figure 1:**
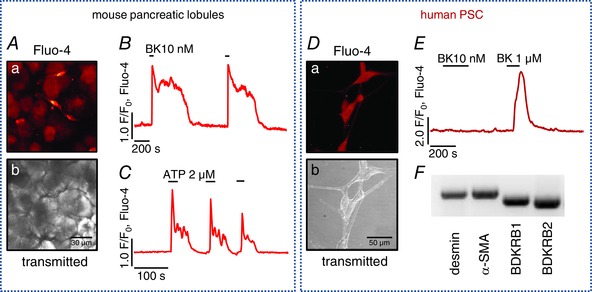
**Morphology and physiological responses of pancreatic stellate cells (PSCs)** *A*, mouse pancreatic lobules: *a*, pancreatic stellate cells (PSCs) stain avidly with the Ca^2+^‐sensitive dye Fluo‐4; *b*, transmitted light image of *a*. *B*, typical Ca^2+^ response to 10 nm bradykinin (BK) in mouse PSCs (*n* = 7). *C*, typical Ca^2+^ response to 2 μm ATP (*n* = 5). *D*, human pancreatic stellate cells (hPSCs) in culture: *a*, hPSCs stained with Fluo‐4; *b*, transmitted light image of *a*. *E*, typical responses to bradykinin (BK) in hPSCs (*n* = 12): 1 μm BK induces a Ca^2+^ transient, whereas 10 nm does not. *F*, mRNA expression of desmin, α‐smooth muscle actin, bradykinin receptor B1 and B2 in hPSCs.

Although the exocrine pancreas mainly consists of pancreatic acinar cells (PACs), other less conspicuous cell types, such as pancreatic stellate cells (PSCs), are woven into the tissue. Two experimental models were used in this study: (1) pancreatic lobules isolated from the mouse pancreas; (2) and primary pancreatic stellate cells of human origin (hPSCs). Pancreatic lobules are a perfect *ex vivo* model, closely resembling the native environment of the pancreas, which allows for investigation of signalling events simultaneously induced in different cell types. Figure 1 *A* shows staining of a pancreatic lobule with the Ca^2+^‐sensitive dye Fluo‐4 AM, where PSCs exhibit a stronger signal as compared to the surrounding PACs. ATP at micromolar concentrations and bradykinin (BK) at low nanomolar concentrations are known to induce elevations in the cytosolic [Ca^2+^] ([Ca^2+^]_i_) of PSCs (Gryshchenko *et al*. 2016 *a*). Figure 1 *B* and *C* demonstrate typical [Ca^2+^]_i_ elevations recorded in PSCs upon stimulation with 10 nm BK and 2 μm ATP, respectively. BK consistently induced biphasic responses: an initial large cytosolic Ca^2+^ transient followed by a sustained Ca^2+^ plateau caused by Ca^2+^ entry (Gryshchenko *et al*. 2016 *a*). Since BK induces Ca^2+^ rises in PSCs but not in PACs, acute Ca^2+^ signal generation in response to BK was frequently used in this study to verify the stellate phenotype in cell clusters.

hPSCs stained with the Ca^2+^‐sensitive dye Fluo‐4 AM are depicted in Fig. 1 *D*. BK at 1 μm, but not 10 nm, induced a single cytosolic Ca^2+^ transient without a sustained plateau phase (Fig. 1 *E*), which is similar to what has previously been shown for PSCs in culture (Won *et al*. 2011). Despite the low sensitivity to BK, hPSCs express both bradykinin receptor types 1 and 2 (BDKRB1 and BDKRB2), as shown in Fig. 1 *F*. Desmin is one of the markers for stellate cells (Apte *et al*. 1998) and the presence of α‐smooth muscle actin (α‐SMA) indicates that hPSCs in culture already acquired their activated phenotype (Fig. 1 *F*).

### PSCs respond to bile acids

Acute biliary pancreatitis is initiated by bile reflux into the pancreas. In isolated PACs, bile acids cause global [Ca^2+^]_i_ elevations via intracellular store depletion and subsequent Ca^2+^ entry (Voronina *et al*. 2002; Lau *et al*. 2005). Until now the effects of bile acids on PSCs have not been tested. Since PSCs also reside in the exocrine pancreas and their fate is tightly intertwined with that of PACs, it becomes important to understand whether PSCs are sensitive to pathophysiological stimuli and if so, whether that might play a part in the induction of acute biliary pancreatitis.

**Figure 2:**
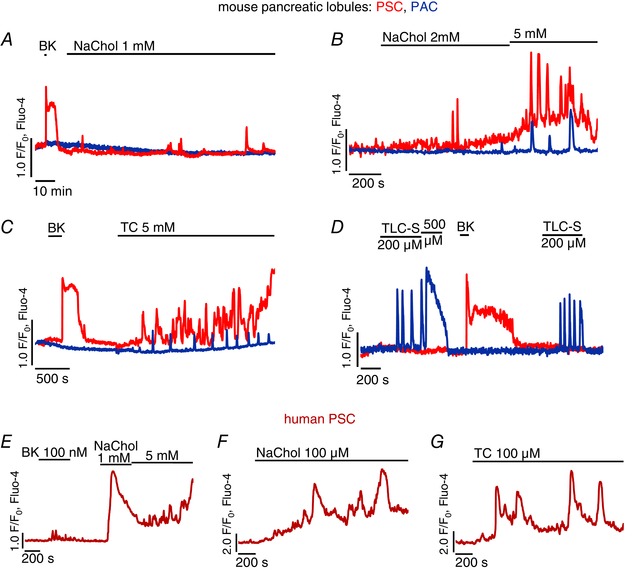
**Ca^2+^ responses to bile acids in pancreatic stellate cells (PSCs)** *A–D*, typical Ca^2+^ responses to bile acids recorded simultaneously in pancreatic stellate cells (PSCs, red traces) and pancreatic acinar cells (PACs, blue traces) in pancreatic lobules. Responses to 10 nm bradykinin (BK) were used as a marker of PSCs. The bile acid salts are as follows: *A*, 1 mm sodium cholate, 2 h treatment (*n*_PSC_ = 4, *n*_PAC_ = 3); *B*, 2 mm and 5 mm sodium cholate (*n*_PSC_ = 10, *n*_PAC_ = 9); *C*, 5 mm taurocholate (*n*_PSC_ = 4, *n*_PAC_ = 6); *D*, 200 μm and 500 μm taurolithocholic acid 3‐sulfate (*n*_PSC_ = 5, *n*_PAC_ = 14). *E–G*, typical Ca^2+^ responses to bile acids recorded in hPSCs: *E*, 1 mm and 5 mm sodium cholate after 100 nm bradykinin (*n* = 12); *F*, 100 μm sodium cholate (*n* = 10); *G*, 100 μm taurocholate (*n* = 37).

Here, we tested the effects of three bile acid salts, sodium cholate (NaChol), taurocholate (TC), and taurolithocholic acid 3‐sulfate (TLC‐S), on Fluo‐4‐loaded pancreatic lobules. Figure 2 *A* shows that 1 mm NaChol caused irregular and small [Ca^2+^]_i_ oscillations in PSCs, whereas effects on PACs were absent. At higher concentrations, 2 mm and 5 mm, NaChol induced global [Ca^2+^]_i_ elevations in all tested PSCs but only very infrequent Ca^2+^ spikes in PACs (Fig. 2 *B*). TC (5 mm) also caused Ca^2+^ signals in PSCs and only modest oscillations in PACs (Fig. 2 *C*). In contrast, TLC‐S at micromolar concentrations has already been demonstrated to trigger [Ca^2+^]_i_ elevations and cell death in PACs and to activate Ca^2+^‐independent cationic currents (Voronina *et al*. 2005). Here, 200 μm and 500 μm TLC‐S exerted the opposite effect to that of NaChol and TC (Fig. 2 *D*), inducing robust Ca^2+^ oscillations in PACs, but not in PSCs. The latter showed typical responses to 10 nm BK.

Similarly, 1 mm and 5 mm NaChol caused rapid and global [Ca^2+^]_i_ elevations in hPSCs, whereas 100 nm BK induced only very modest oscillations (Fig. 2 *E*). Treatment with 100 μm NaChol or TC, a ten times lower concentration than the lowest concentration capable of triggering at least a minor [Ca^2+^]_i_ elevation in pancreatic lobules, was sufficient for induction of substantial [Ca^2+^]_i_ elevations in hPSCs (Fig. 2 *F* and *G*).

### Responses to bile acids in PSCs are dependent on extracellular Ca^2+^

**Figure 3:**
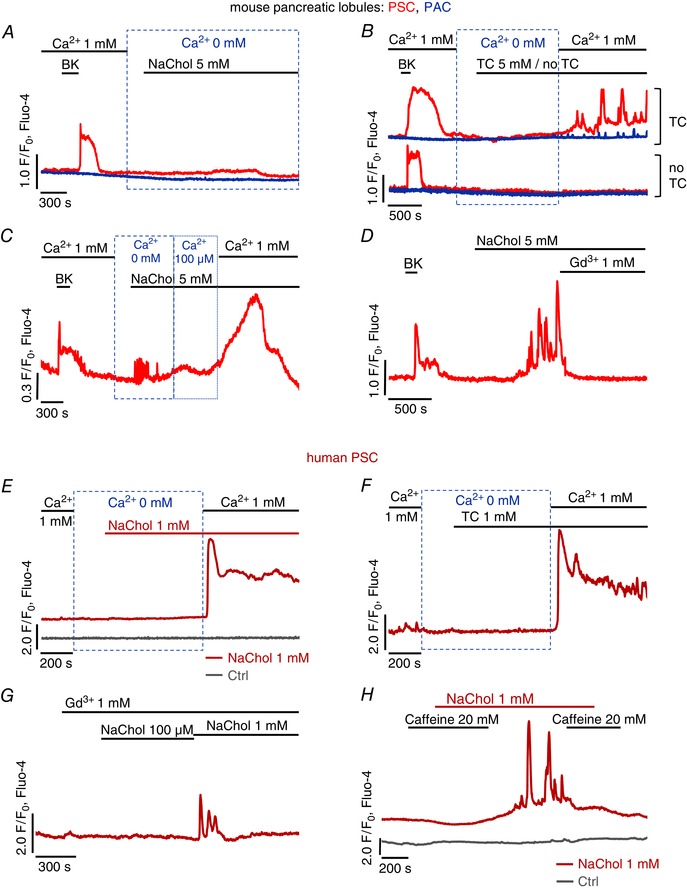
**Dependence of bile acid‐induced responses on extracellular Ca^2+^** *A–D*, typical Ca^2+^ responses to bile acids in the presence and absence of external Ca^2+^ recorded simultaneously in neighbouring pancreatic stellate cells (PSCs, red traces) and pancreatic acinar cells (PACs, blue traces) in pancreatic lobules. *A*, responses to 5 mm sodium cholate in the absence of extracellular Ca^2+^ (*n*_PSC_ = 20, *n*_PAC_ = 22). *B*, responses to 5 mm taurocholate in the absence of extracellular Ca^2+^ followed by application of 1 mm Ca^2+^ (upper traces, *n*_PSC_ = 10, *n*_PAC_ = 10); control traces (lower) show that addition of 1mm Ca^2+^ to untreated cells does not trigger responses *(n*_PSC_ = 6, *n*_PAC_ = 7); 10 nm bradykinin was applied at the beginning as confirmation of the stellate phenotype. *C*, small response of a PSC to 5 mm sodium cholate in the absence of extracellular Ca^2+^, followed by application of 100 μm Ca^2+^ and then 1 mm Ca^2+^ (*n* = 4). *D*, typical response of a PSC to 5 mm cholate in the presence of 1 mm extracellular Ca^2+^ followed by application of 1 mm Gd^3+^ (*n* = 8). *E–H*, typical Ca^2+^ responses recorded in hPSCs to cholate and taurocholate in the absence of Ca^2+^, cholate in the presence of Gd^3+^, and cholate ± caffeine. *E*, 1 mm cholate in the absence of extracellular Ca^2+^ followed by application of 1 mm Ca^2+^ (red trace, *n* = 9); readmission of extracellular Ca^2+^ to untreated cells does not trigger responses (grey trace, *n* = 4). *F*, 1 mm taurocholate in the absence of extracellular Ca^2+^ followed by an addition of 1 mm Ca^2+^ (*n* = 8). *G*, typical Ca^2+^ response to 100 μm and 1 mm sodium cholate in the presence of 1 mm Gd^3+^ (*n* = 11). *H*, typical Ca^2+^ response to 1 mm cholate in the presence and absence of 20 mm caffeine (red trace, *n* = 9); control trace (grey) shows that application of caffeine alone does not trigger any responses (*n* = 6).

Previous reports have demonstrated that certain bile acids empty intracellular stores triggering Ca^2+^ entry in PACs (Voronina *et al*. 2002; Lau *et al*. 2005). To test this in PSCs, pancreatic lobules containing PACs and PSCs were treated with 5 mm NaChol (Fig. 3 *A*) or 5 mm TC (Fig. 3 *B*) in the absence of extracellular Ca^2+^. Apart from small oscillations (as seen in Fig. 3 *C*) bile acids consistently failed to induce marked increases of [Ca^2+^]_i_ in either cell type. Upon readmission of extracellular Ca^2+^ (1 mm), PSCs developed global [Ca^2+^]_i_ elevations, whereas only very modest oscillations were seen in PACs (Fig. 3 *B*, upper traces). Readmission of Ca^2+^ to untreated cells did not cause any responses (Fig. 3 *B*, lower traces). Similarly, removal of extracellular Ca^2+^ blocked responses to 1 mm NaChol (Fig. 3 *E*) and 1 mm TC (Fig. 3 *F*) in hPSCs and readmission of extracellular Ca^2+^ recovered the responses.

It seemed possible that the detergent properties of the bile acids (Linke, 2009) could compromise the integrity of the plasma membrane and that such an effect might explain the cytosolic Ca^2+^ elevations, particularly since PSCs did not develop global Ca^2+^ responses to 5 mm NaChol in the absence of external Ca^2+^. However, at an extracellular [Ca^2+^] of 100 μm, which does not efficiently support Ca^2+^ influx via store‐operated channels (Peel *et al*. 2008), NaChol only caused a very modest increase, if any, in [Ca^2+^]_i_, a response that was very much smaller than that observed after a further increase of the extracellular [Ca^2+^] to 1 mm (Fig. 3 *C*). Furthermore, Fig. 3 *D* shows that Ca^2+^ signals elicited by 5 mm NaChol in PSCs were completely inhibited by 1 mm Gd^3+^. Gd^3+^ also blocked responses in hPSCs to 100 μm NaChol (Fig. 3 *G*). In the presence of Gd^3+^, an increase in the NaChol concentration to 1 mm only resulted in short‐lasting Ca^2+^ oscillations followed by a quick return to the resting level (Fig. 3 *G*). Since Gd^3+^ is a non‐specific, but very potent, blocker of Ca^2+^ channels (Bourne & Trifaro, 1982), the complete inhibition of the NaChol‐induced [Ca^2+^]_i_ elevations in PSCs indicates that the responses were dependent on Ca^2+^ influx through plasma membrane Ca^2+^ channels and not due to bile acid‐mediated membrane permeabilisation.

In PACs, it has been shown that bile acids release Ca^2+^ from intracellular stores through activation of inositol triphosphate receptors (IP_3_Rs) and ryanodine receptors (RyRs) (Gerasimenko *et al*. 2006). In the presence of 20 mm caffeine, an inhibitor of IP_3_Rs (Wakui *et al*. 1990; Toescu *et al*. 1992), 1 mm NaChol did not induce cytosolic Ca^2+^ responses (Fig. 3 *H*, red trace). Upon removal of caffeine, NaChol‐elicited signals were restored and subsequent reapplication of caffeine abolished the responses again (Fig. 3 *H*, red trace). Application of 20 mm caffeine alone did not trigger [Ca^2+^]_i_ elevation in hPSCs (Fig. 3 *H*, grey trace).

### Necrosis induced by bile acid salts is dependent on the presence of extracellular Ca^2+^

**Figure 4:**
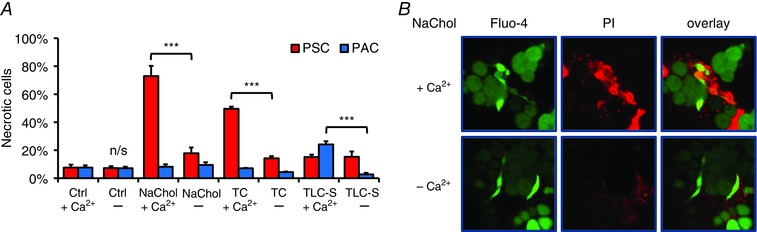
**Bile acid‐induced cell death in pancreatic stellate cells (PSCs)** *A*, necrosis induced by 30 min incubation with a bile acid in the presence or absence of 1 mm Ca^2+^. Red bars represent necrotic pancreatic stellate cells (PSCs), and blue bars represent necrotic pancreatic acinar cells (PACs) in the same lobules. From the left: control 1 mm Ca^2+^, no bile acid (*N* = 4); control no Ca^2+^, no bile acids (*N* = 4); 5 mm cholate, 1 mm Ca^2+^ (*N* = 6); 5 mm cholate, no Ca^2+^ (*N* = 3); 5 mm taurocholate, 1 mm Ca^2+^ (*N* = 4); 5 mm taurocholate, no Ca^2+^ (*N* = 3); 200 μm taurolithocholic acid 3‐sulfate, 1 mm Ca^2+^ (*N* = 6); and 200 μm taurolithocholic acid 3‐sulfate, no Ca^2+^ (*N* = 3). *B*, PSC necrosis is induced by 5 mm cholate in the presence of 1 mm Ca^2+^ (upper panel) but not in the absence of Ca^2+^ (lower panel); from the left: live PSCs (bright green) and PACs (dark green); necrotic cells (red); overlay of the two images.

In order to investigate the pathophysiological consequences of Ca^2+^ signals induced by bile acids in PSCs and their implications for acute pancreatitis, we performed a necrosis assay in which isolated pancreatic lobules were treated for 30 min with one of three bile acid salts: 5 mm NaChol, 5 mm TC or 200 μm TLC‐S in the presence or absence of extracellular Ca^2+^. Figure 4 *A* shows that in the presence of extracellular Ca^2+^, both NaChol and TC caused substantial necrosis in PSCs (73.0 ± 7.3% and 49.6 ± 1.6%, respectively) as compared to control (7.6 ± 2.1%, both *P* < 0.001). In the absence of extracellular Ca^2+^ the levels of NaChol‐ and TC‐elicited necrosis were markedly reduced (17.8 ± 4.2, *P* < 0.001 and 14.2 ± 1.5, *P* < 0.001, respectively; Fig. 4 *A*). Neither NaChol nor TC induced substantial necrosis in PACs in the time frame of 30 min, regardless of whether extracellular Ca^2+^ was present or not (Fig. 4 *A*). In contrast, TLC‐S, in the presence of Ca^2+^, induced necrosis in PACs (24.2 ± 2.2%); and Ca^2+^ removal led to a reduction of TLC‐S‐elicited necrosis in these cells (2.7 ± 1.1%, *P* < 0.001, Fig. 4 *A*). Figure 4 *B* shows representative images of pancreatic lobules treated with NaChol in the presence (top panel) and absence (bottom panel) of extracellular Ca^2+^. PI‐stained necrotic PSCs are seen as bright red circle spots only in the top panel. These results demonstrate that PSC necrosis was a direct downstream effect of pathological Ca^2+^ signals caused by the bile acid salts NaChol and TC. Inhibition of these signals by removal of extracellular Ca^2+^ abolished cell death in PSCs. Further, TLC‐S, which did not trigger Ca^2+^ signals in mouse PSCs, failed to induce necrosis in these cells.

### Responses to bile acids in PSCs are dependent on extracellular Na^+^

**Figure 5:**
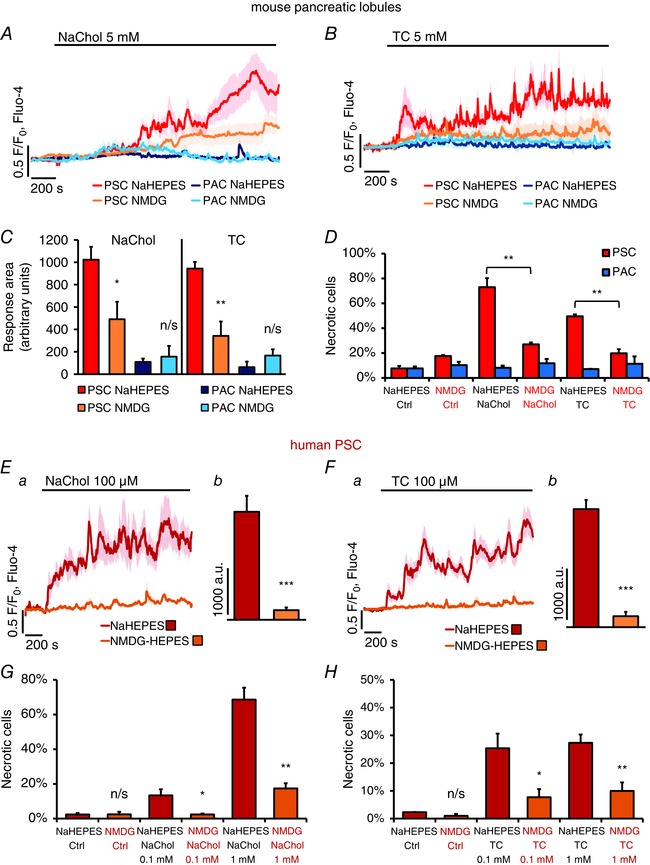
**Dependence of Ca^2+^ responses and necrosis in pancreatic stellate cells (PSCs) on extracellular Na^+^** *A*, average traces (± SEM) showing Ca^2+^ responses to 5 mm sodium cholate in PSCs recorded in NaHepes (red, *n* = 8), PSCs in NMDG‐Hepes (yellow, *n* = 13), PACs in NaHepes (dark blue, *n* = 7) and PACs in NMDG‐Hepes (light blue, *n* = 13). *B*, average traces (± SEM) showing Ca^2+^ responses to 5 mm taurocholate in PSCs recorded in NaHepes buffer (red, *n* = 6), PSCs in NMDG‐Hepes (yellow, *n* = 8), PACs in NaHepes (dark blue, *n* = 18), PACs in NMDG‐Hepes (light blue, *n* = 14). *C*, the responses shown in *A* and *B* were quantitatively analysed by comparing the average Ca^2+^ increase above the baseline levels recorded between 200 and 2000 s (*n* numbers as in *A* and *B*). *D*, the bar chart compares necrosis in mouse PSCs (red bars) and PACs (blue bars) induced by 30 min incubation in the presence of 5 mm cholate or 5 mm taurocholate in NaHepes (bars also shown in Fig. 4 *A*) or in NMDG‐Hepes (*N*_Ctrl_ = 4, *N*_NaChol_ = 3, *N*_TC_ = 3). *Ea*, average traces (± SEM) showing cytosolic Ca^2+^ responses to 100 μm cholate in hPSCs in NaHepes (red trace, *n* = 10) and in NMDG‐Hepes (orange trace, *n* = 11); *Eb*, average Ca^2+^ responses above the baseline levels (arbitrary units) calculated between 200 and 2000 s for the individual traces averaged in *Ea*. *Fa*, average traces (± SEM) showing cytosolic Ca^2+^ responses to 100 μm taurocholate in hPSCs in NaHepes (red trace, *n* = 37) and in NMDG‐Hepes (orange trace, *n* = 49); *Fb*, average Ca^2+^ responses above the baseline levels (arbitrary units) calculated between 200 and 2000 s for the individual traces averaged in *Fa*. *G*, the bar chart shows necrosis induced in hPSCs by 2 h incubation with 0.1 and 1 mm cholate, both in the presence (NaHepes) or absence of extracellular Na^+^ (NMDG‐Hepes); *N* = 4 for all. *H*, necrosis induced in hPSCs by 2 h incubation with 0.1 and 1 mm taurocholate, both in the presence (NaHepes) or absence of extracellular Na^+^ (NMDG‐Hepes); *N* = 4 for all.

Cellular uptake of bile acids is generally facilitated by specific membrane transporters utilising either Na^+^‐dependent or ‐independent mechanisms (Trauner & Boyer, 2003; Dawson *et al*. 2009). To test whether bile acid uptake and therefore the effects on PSCs are dependent on Na^+^, bile acid‐induced Ca^2+^ signals in pancreatic lobules were compared in two different conditions: in normal extracellular NaHepes and in a buffer that contained *N*‐methyl‐d‐glucamine (NMDG^+^) instead of Na^+^ (NMDG‐Hepes). Since cholate and taurocholate were applied as sodium salts, the removal of external Na^+^ was not complete, but the extracellular Na^+^ concentration was effectively reduced from 140 mm to 5 mm. In PSCs, replacement of extracellular Na^+^ with NMDG^+^ reduced very markedly the magnitude of the [Ca^2+^]_i_ elevations elicited by NaChol (Fig. 5 *A*) or TC (Fig. 5 *B*), whereas in PACs the tiny Ca^2+^ oscillations evoked by these bile acids were similar in the presence and absence of extracellular Na^+^ (Fig. 5 *A* and *B*). In order to quantitatively compare the responses, the area between 200 and 2000 s under each individual trace was calculated and the average values were compared in Fig. 5 *C*. In the absence of extracellular Na^+^, the response areas calculated for NaChol‐ or TC‐treated PSCs were significantly smaller than those of PSCs in normal NaHepes (1023 ± 115 a.u. *versus* 491 ± 157 a.u., *P* = 0.013; and 944 ± 59 a.u. *versus* 342 ± 129 a.u., *P* = 0.002, Fig. 5 *C*).

Figure 5 *D* shows the extent of cell necrosis induced by 30 min incubation of pancreatic lobules with 5 mm NaChol or 5 mm TC in NMDG‐Hepes and compares these necrosis levels with those induced in normal NaHepes (also shown in Fig. 4 *A*). Even though the absence of Na^+^ was associated with slightly higher baseline levels of necrosis in PSCs (*P* = 0.011), there was a substantial decrease in the degree of PSC necrosis induced by NaChol (from 73.0 ± 7.3% to 27.0 ± 1.6%, *P* = 0.001) or TC (from 49.6 ± 1.6% to 19.8 ± 3.3%, *P* = 0.004) in NMDG‐Hepes compared to the same concentration of the bile acid in NaHepes. PAC necrosis remained at the same level in all tested samples.

In hPSCs, Ca^2+^ signals induced by 100 μm NaChol (Fig. 5 *Ea*) or 100 μm TC (Fig. 5 *Fa*), were abolished when NaHepes was replaced by NMDG‐Hepes. This is reflected by a significant difference in average areas calculated between 200 and 2000 s under individual traces (2291 ± 341 a.u. *versus* 203 ± 57 a.u., *P* < 0.001, Fig. 5 *Eb*; and 2139 ± 165 a.u. *versus* 199 ± 79 a.u., *P* < 0.001, Fig. 5 *Fb*). Figures 5 *G* and *H* show cell death in hPSCs exposed for 2 h to bile acid salts in the presence or absence of extracellular Na^+^. Treatment with 0.1 mm NaChol triggered low levels of necrosis (13.4 ± 3.5%), which was abolished by substitution of Na^+^ with NMDG^+^ (2.4 ± 0.5%, *P* = 0.021, Fig. 5 *G*). At the higher concentration of 1 mm, NaChol induced significantly more necrosis in NaHepes buffer (68.6 ± 6.8%) than in NMDG‐Hepes (17.4 ± 3.0%, *P* = 0.002, Fig. 5 *G*). TC induced comparable levels of necrosis at 0.1 and 1 mm, when applied in NaHepes buffer (25.3 ± 5.3% and 27.3 ± 3.0%), and these effects were markedly decreased upon removal of extracellular Na^+^ (7.7 ± 2.9%, *P* = 0.036 and 10.0 ± 3.1%, *P* = 0.007, respectively, Fig. 5 *H*). Taken together, these results show that PSCs not only utilise Na^+^‐dependent transport of bile acids, but that these mechanisms play significant roles in their pathophysiology.

### PSCs express NTCP

**Figure 6:**
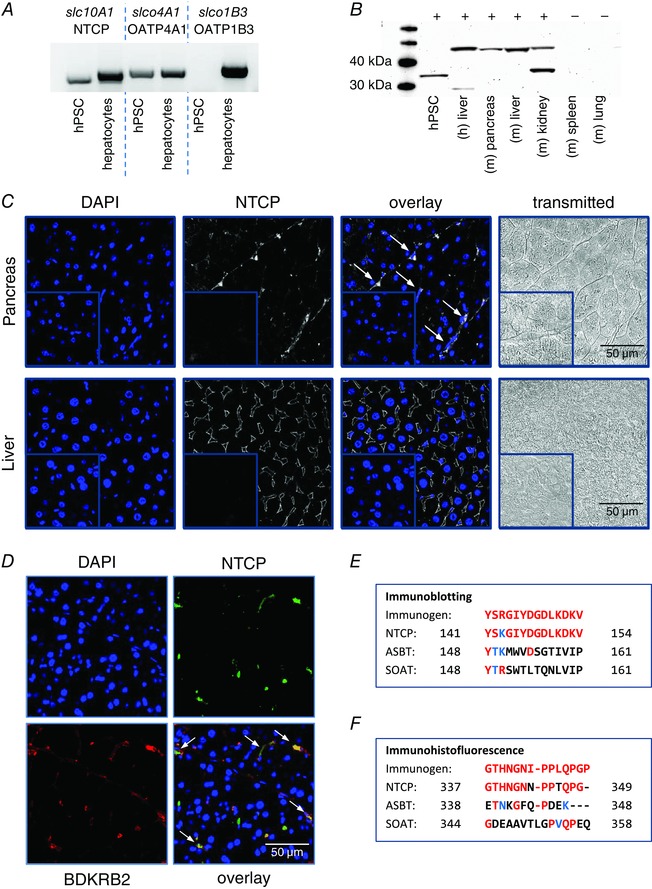
**Expression of NTCP in pancreatic stellate cells (PSCs)** *A*, mRNA expression of selected genes encoding bile acid transporters in human pancreatic stellate cells (hPSCs) and human hepatocytes. PCR products for the sodium–taurocholate cotransporting polypeptide (NTCP, *slc10A1)* and sodium independent transporters, OATP4A1 (*slco4A1*) and OATP1B3 *(slco1B3)*, are present in human hepatocytes; hPSCs express *slc10A1* and *slco4A1*, but not *slco1B3*. *B*, immunoblotting shows expression of NTCP in different tissues; lines from the left: mass marker, hPSC, four positive controls (human liver and mouse pancreas, liver and kidney), two negative controls (mouse spleen and lung). *C*, immunohistofluorescence (IHF) staining for NTCP in fixed mouse tissue sections: the pancreas (upper panel) and the liver (lower panel). From the left: DAPI (blue), NTCP (white), overlay images (white arrows indicate PSCs), and transmitted light images. Insets show corresponding staining in controls without primary antibody. *D*, double IHF staining for NTCP and the bradykinin receptor B2 (BDKRB2). From the upper left: DAPI (blue), NTCP (green), BDKRB2 (red), fluorescence overlay (PSCs are indicated by white arrows). *E* and *F*, the comparison of homological sequences of three Na^+^‐dependent mouse transporters (NTCP, ASBT and SOAT) with immunogens used for generation of the antibodies for immunoblotting (*E*) and IHF (*F*). Red letters represent amino acids identical to those in the immunogen sequence; blue letters are similar amino acids, and black letters show amino acids that are different. Only mouse NTCP sequences share high sequence identity with the immunogens.

The effects of bile acids on PSCs could be explained by the transport characteristics and the substrate specificity of the sodium–taurocholate cotransporting polypeptide (NTCP). We therefore attempted to probe for NTCP at mRNA and protein levels in PSCs. cDNA, generated from mRNA of hPSCs and human hepatocytes, was amplified in a PCR reaction with primers designed for human NTCP (*slc10A1*) and two Na^+^‐independent transporters for comparison: OATP4A1 (*slco4A1*) and OATP1B3 (*slco1B3*). hPSCs express detectable levels of NTCP and OATP4A1, but not OATP1B3 (Fig. 6 *A*).

Immunoblotting detected NTCP in the total protein extracts from hPSCs, human liver, mouse pancreas, liver and kidney, but not in the negative controls from mouse spleen and lung (Fig. 6 *B*). NTCP is a glycoprotein with an apparent molecular weight of 50 kDa (fully glycosylated) or 33.5 kDa (not glycosylated) (Ananthanarayanan *et al*. 1994; Stieger *et al*. 1994).

Finally, we performed immunohistofluorescence staining (IHF) for NTCP on paraffin‐fixed sections of the mouse pancreas and liver (Fig. 6 *C*). The liver tissue was used as a positive control. The upper panel in Fig. 6 *C* shows that NTCP (white) is expressed in mouse PSCs as string‐like structures in between pancreatic acini. The fluorescence signal is accumulated close to the elongated nuclei of PSCs, but is not present in PACs. The lower panel shows a very specific diamond‐shaped pattern of NTCP at the basolateral domain of mouse hepatocytes, which is in line with previous reports (Ananthanarayanan *et al*. 1994; Keane *et al*. 2007). Insets demonstrate negative control staining of the pancreatic and liver tissue (no primary antibody). Figure 6 *D* shows a double immunostaining of mouse pancreatic tissue for NTCP (green) and the bradykinin receptor type 2 (BDKRB2) (red). The latter is present in PSCs, but not in PACs, and thus is used here as a cell type marker. The overlay image shows that the NTCP signal mostly co‐localises with BDKRB2, indicating that both proteins are expressed in PSCs (white arrows).

The SLC10 protein family also contains at least two other Na^+^‐dependent transporters: apical sodium‐dependent bile acid transporter (ASBT) and sodium‐dependent organic anion transporter (SOAT). The polyclonal antibody used for immunoblotting was designed to bind a fragment in the middle region of human NTCP (141–154 amino acids): YSRGIYDGDLKDKV. Figure 6 *E* shows a comparison of the immunogen to the homological sequences of Na^+^‐dependent transporters belonging to the SLC10 family in mouse. Only mouse NTCP shows high sequence identity (93%) to the immunogen, whereas ASBT and SOAT do not, and thus it is unlikely that they serve as epitopes for the antibody. The polyclonal antibody used for IHF was generated with a 14‐amino acid peptide GTHNGNIPPLQPGP, corresponding to the C‐terminal end (339–352) of rat NTCP (Ananthanarayanan *et al*. 1994), which shares high sequence identity with mouse NTCP (85%), but not ASBT or SOAT (Fig. 6 *F*). It is therefore expected to specifically bind mouse NTCP, but not other members of the SLC10 family.

### PSCs affect the fate of neighbouring PACs

**Figure 7:**
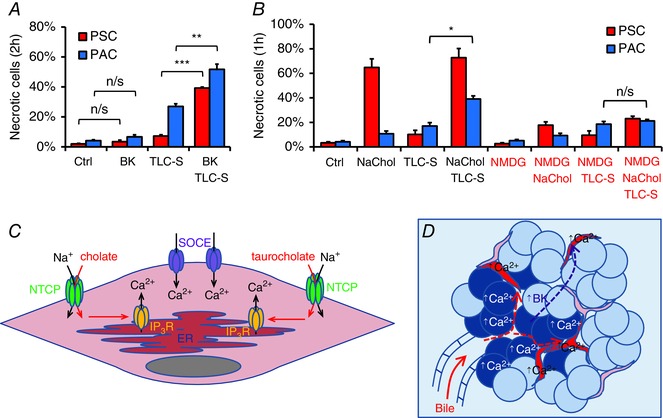
**Escalated effects of bile acids in pancreatic lobules** *A*, necrosis in mouse PSCs (red) and PACs (blue) treated for 2 h with 10 nm bradykinin (BK), 200 μm taurolithocholic acid 3‐sulfate (TLC‐S) or both (*N* = 4 for all groups). *B*, necrosis induced in mouse PSCs (red) and PACs (blue) during 1 h incubation with 5 mm sodium cholate (NaChol), 200 μm taurolithocholic acid 3‐sulfate (TLC‐S) and both bile acids, all in the presence (NaHepes) or absence of extracellular Na^+^ (NMDG‐Hepes) (*N* = 3 for all groups). *C*, proposed mechanism of bile acid‐induced Ca^2+^ signals in a PSC: bile acids (cholate and taurocholate) enter PSCs via membrane transporters, such as NTCP, and induce IP_3_R‐mediated Ca^2+^ release from the ER; depletion of the ER triggers opening of store‐operated Ca^2+^ entry (SOCE) channels leading to [Ca^2+^]_i_ overload. *D*, schematic illustration of Ca^2+^ events in acute biliary pancreatitis: concentrated bile reaches pancreatic lobules and triggers excessive Ca^2+^ responses in PACs (blue) and PSCs (red) followed by necrosis; damage to PACs leads to generation of bradykinin, which, in turn, induces Ca^2+^ responses in PSCs making them more susceptible to bile‐elicited damage.

Damage to the pancreatic tissue is associated with inflammation and BK is an inflammatory mediator that, at pathophysiologically relevant concentrations, induces Ca^2+^ signals in PSCs, but not in PACs (Gryshchenko *et al*. 2016 *a*). BK is generated from kininogens by enzymatic cleavage by kallikreins, which also happen to be stored in the zymogen granules of PACs (Schachter, 1969). Therefore continued damage to PACs triggers the release of proteases and generates BK (Orlov & Belyakov, 1978), which can act on PSCs (Gryshchenko *et al*. 2016 *b*). There is no direct transfer of Ca^2+^ signals from PSCs to PACs (Gryshchenko *et al*. 2016 *a*), but PSCs could exert effects on PACs via different types of interaction and thus contribute to the pathogenesis of pancreatitis. Figure 7 *A* shows the extent of necrotic cell death induced by 2 h treatment with 200 μm TLC‐S in the presence of 10 nm BK. BK alone neither induced necrosis in PSCs (*P* = 0.25) nor in PACs (*P* = 0.15) when compared to the control; and 200 μm TLC‐S applied alone only slightly increased necrosis in PSCs (7.2 ± 0.9% *versus* 2.0 ± 0.4%, *P* = 0.005) predominantly killing PACs (27.0 ± 1.8% *versus* 4.1 ± 0.6%, *P* < 0.001). However, addition of BK to the TLC‐S treatment caused significantly more necrosis both in PSCs (39.2 ± 0.7%, *P* < 0.001) and in PACs (51.7 ± 3.5%, *P* = 0.002) as compared to TLC‐S alone. This shows that relatively modest effects of TLC‐S on PSCs can be dramatically enhanced in the presence of BK. Intensified necrosis of PSCs could further increase PAC death.

Bile reflux leads to exposure of the pancreas to a concentrated mixture of bile acids, not just a single component. Figure 7 *B* shows that combined treatment for 1 h with 5 mm NaChol and 200 μm TLC‐S caused substantial necrosis in PSCs (72.8 ± 7.5%), similar to that elicited by NaChol alone (64.8 ± 7.0%, *P* = 0.48); and markedly increased necrosis in PACs as compared to TLC‐S alone (39.1 ± 2.5% *versus* 17.0 ± 2.7%, *P* = 0.004). Inhibition of Na^+^‐dependent uptake of NaChol in PSCs (NMDG^+^) resulted in a marked reduction of NaChol‐induced necrosis in PSCs (17.7 ± 2.7%, *P* = 0.013). However, while neither NaChol‐ nor TLC‐S‐induced cell death in PACs was affected by removal of Na^+^ (*P* = 0.63 and *P* = 0.69, respectively), combined treatment with TLC‐S and NaChol no longer induced more necrosis in PACs than TLC‐S alone (21.2 ± 1.2% *versus* 18.5 ± 2.2%, *P* = 0.37). Lack of the exacerbated necrosis in PACs in the absence of Na^+^ may be attributed to a much smaller proportion of adjacent PSCs killed by NaChol (23.1 ± 1.9%, *P* = 0.017), as compared to the level seen in the presence of Na^+^ (72.8 ± 7.5%). These results suggest that necrosis induced in pancreatic lobules depends on the effects of bile acids on both PACs and PSCs. Extensive necrosis in PSCs may further sensitise PACs to bile acid‐induced killing.

## Discussion

This study demonstrates for the first time that not only acinar cells (PACs) but also stellate cells (PSCs) are important targets for the action of bile acids in biliary acute pancreatitis. Even though PACs and PSCs are located in close proximity in pancreatic lobules they respond independently to pathophysiological stimuli and display very different sensitivities. Some of the naturally occurring bile acid salts, NaChol and TC, elicit dramatic Ca^2+^ signals in PSCs followed by necrotic death, but have very little effects on the neighbouring PACs. Another bile acid, TLC‐S, acts primarily on PACs and only to a lesser extent on PSCs. Although PACs are known to respond to NaChol and TC when isolated as single cells or small clusters (Kim *et al*. 2002), we demonstrate here that they are more resistant to pathological stimuli when incorporated in their natural environment of pancreatic lobules.

PSCs are different from PACs in many respects. They are relatively small with a big centrally located nucleus and their intracellular Ca^2+^ stores may therefore be smaller than those of PACs. Thus the development of large Ca^2+^ signals requires influx of Ca^2+^ from the extracellular space. Therefore, it is not surprising that removal of Ca^2+^ from the extracellular buffer completely abolished both Ca^2+^ signals and necrosis in PSCs (Figs 3 *A* and *B* and 4 *A*). Since inhibition of bile acid‐induced Ca^2+^ signals by caffeine (Fig. 3 *H*) indicates involvement of IP_3_Rs and the ER, we postulate that bile acid salts, upon entering PSCs, activate intracellular Ca^2+^ release through IP_3_Rs. A very similar mechanism was already proposed to explain TLC‐S‐induced Ca^2+^ signals in PACs (Voronina *et al*. 2002); and bile acid‐mediated mobilisation of Ca^2+^ via IP_3_Rs was also observed in human colonic crypts (Pallagi‐Kunstar *et al*. 2015). In PSCs, however, it would appear that the initial release of Ca^2+^ from the intracellular stores very quickly leads to depletion of the ER, which triggers opening of store‐operated Ca^2+^ entry (SOCE) channels in the plasma membrane (Parekh, 2006, 2007) and influx of Ca^2+^ into the cytosol (Fig. 7 *C*). The importance and the magnitude of Ca^2+^ entry in these cells is also reflected by the biphasic response to BK (Fig. 1 *B*), where the initial short‐lasting Ca^2+^ transient is always followed by a prolonged elevated [Ca^2+^]_i_ plateau caused by Ca^2+^ entry (Gryshchenko *et al*. 2016 *a*).

Bile is produced by the liver and then flows through the hepatic duct into the gallbladder, where it is stored and concentrated by reabsorption of water. In previous studies bile acid concentrations in the gallbladder bile were found to be in the range of 15.1–272.8 mm (Shiffman *et al*. 1990) or 51.5–246 mm (Keulemans *et al*. 1998), which was substantially higher than in the hepatic bile (7.4–74 mm) (Keulemans *et al*. 1998). To the best of our knowledge, there are no data about the bile acid concentrations present in the pancreas during gallstone‐induced bile reflux. However, given the known values for the gallbladder bile (Shiffman *et al*. 1990; Keulemans *et al*. 1998), it is likely that the pancreatic tissue becomes exposed to bile acids at millimolar concentrations.

Bile acid salts in high millimolar concentrations have detergent properties and are effective membrane permeabilising agents, often used as components of cell lysis buffers (Linke, 2009). Therefore it was necessary to test whether the responses seen in our experiments could be explained by a bile acid‐induced loss of plasma membrane integrity followed by a non‐specific influx of Ca^2+^ to the cytosol. It could even be hypothesised that glycocalyx‐covered PACs (Jonas *et al*. 1986) in lobules are more resistant to the detergent effects of bile acids than unprotected PSCs, hence the increased severity of the effects on the latter. However, we observed no loss of fluorescent Ca^2+^ indicators from the cytosol of PSCs or PACs, which suggests that the detergent effect of bile acids in our experiments was minimal. Furthermore, at an extracellular Ca^2+^ of 100 μm, bile acids hardly increased [Ca^2+^]_i_ in PSCs (Fig. 3 *C*). If the cells had been permeabilised by the bile acids, [Ca^2+^]_i_ should have increased dramatically (in fact to 100 μm). Finally, Gd^3+^ abolished Ca^2+^ signal generation (Fig. 3 *D* and *G*), which indicates that Ca^2+^ influx occurs via ion channels. Therefore it is unlikely that the effects of bile acids on [Ca^2+^]_i_ in PSCs were caused by their detergent properties.

It is well established that certain cell types, such as hepatocytes, cholangiocytes, intestinal enterocytes and the proximal renal tubular cells, can take up bile acids via transporters that utilise mechanisms either dependent on or independent of the Na^+^ gradient across the plasma membrane (Trauner & Boyer, 2003; Dawson *et al*. 2009; Claro da Silva *et al*. 2013). If the effects of NaChol and TC are due to actions inside the cells then specific Na^+^‐dependent or independent mechanisms for bile acid uptake must exist. Our data show that the bile acid effects are dramatically reduced by omission of Na^+^ from the extracellular fluid indicating that Na^+^‐dependent transport of bile acids is a substantial contributor to bile acid uptake in PSCs.

Na^+^‐dependent influx of bile acids is mediated by three known members of the solute carrier family (SLC): (1) the sodium–taurocholate cotransporting polypeptide (NTCP, SLC10A1), predominantly present in hepatocytes; (2) the apical sodium‐dependent bile acid transporter (ASBT, SLC10A2, also referred as ISBT or NTCP2), which is expressed in the intestine; (3) and the sodium‐dependent organic anion transporter (SOAT, SLC10A6). SOAT, whose expression was found to be relatively high in the pancreas, is able to transport sulfo‐conjugated bile acids such as TLC‐S (Geyer *et al*. 2007), which could explain the sensitivity of PACs to this bile acid. In contrast, NTCP mediates the influx of glycine and taurine‐conjugated bile acids as well as, to a lesser extent, unconjugated bile acids (Boyer *et al*. 1994; Hagenbuch & Meier, 1994; Kramer *et al*. 1999; Hata *et al*. 2003). It was previously reported that NTCP is present in the rat pancreas and may facilitate bile acid uptake in PACs (Kim *et al*. 2002). In this study we used the same antibody for IHF and found NTCP to be expressed in mouse PSCs but not in mouse PACs (Fig. 6 *C* and *D*), which could indicate differences in expression of NTCP between different species.

Since NTCP serves as an entry receptor for hepatitis B virus (HBV) (Watashi *et al*. 2014; Yan & Li, 2015; Witt‐Kehati *et al*. 2016), it is already a very attractive target for development of specific drugs, such as Myrcludex B, a lipopeptide which blocks HBV docking to NTCP (Volz *et al*. 2013; Lempp & Urban, 2014; Urban *et al*. 2014). Temporary and reversible inhibition of Na^+^‐dependent bile acid transport in the pancreas could provide a pharmacological tool useful in the acute phase of bile‐induced pancreatitis.

Na^+^‐independent mechanisms most likely play a lesser role in bile acid transport across the PSC membrane. Our data indicate that hPSCs express OATP4A1 (Fig. 6 *A*). The presence of many other transporters has not been tested. However, the detailed molecular characterisation of membrane transport in PSCs is outside the scope of this work.

Our findings also shed new light on the current understanding of the mechanisms promoting pancreatic pathology, which involve not only signalling in PACs but also in PSCs. Although these two cell types have developed independent Ca^2+^ responses to external stimuli and there is no evidence for propagation of Ca^2+^ signals from one cell type to another, we found that induction of Ca^2+^ signals in PSCs by BK makes them susceptible to TLC‐S treatment and increases cell necrosis in PSCs as well as in neighbouring PACs (Fig. 7 *A*). An important role for BK in the development of acute pancreatitis was proposed over half a century ago (Ryan *et al*. 1964) and studies have demonstrated that pharmacological inhibition of bradykinin receptor B2 (BDKRB2) suppresses the histopathological changes of the rat pancreas in a model of biliary acute pancreatitis (Hirata *et al*. 2002) as well as in caerulein‐induced pancreatitis (Griesbacher *et al*. 1993). Therefore, we propose that bile acids upon entering the pancreas induce Ca^2+^ overload in PACs and PSCs, followed by cell necrosis (Fig. 7 *D*). Liberation of enzymes from PACs, including kallikreins, would generate increased levels of BK in the pancreatic tissue. In turn, BK, by inducing Ca^2+^ signals in PSCs, would further escalate the pathological effects of bile acids in the pancreas.

Finally, the fact that PSCs are very sensitive targets for bile acids might have other important implications for acute biliary pancreatitis. PSCs are responsible for development of tissue fibrosis and morphological features present in chronic injury of the pancreas (Haber *et al*. 1999; Casini *et al*. 2000; Neuschwander‐Tetri *et al*. 2000). As opposed to alcohol‐induced pancreatitis, fibrosis is a less common complication of acute biliary pancreatitis (Pareja *et al*. 2003; Bertilsson *et al*. 2015; Ahmed Ali *et al*. 2016). Our study provides a plausible explanation for this observation. Since acute exposure to bile acids is likely to kill PSCs, the decrease in their numbers could potentially diminish formation of excessive tissue fibrosis during the repair processes of the pancreas.

## Additional information

### Competing interests

The authors declare no competing interests.

### Author contributions

All experiments were performed in the MRC Group laboratory, School of Biosciences, Cardiff University. O.H.P., O.V.G. and J.V.G. conceived and supervised the project. P.E.F. designed and conducted Ca^2+^ measurements; P.E.F. and M.A.J. did molecular biology and histological experiments. P.E.F. performed data analysis. All authors took part in discussion and interpretation of data. The manuscript was written by P.E.F. and O.H.P., revised and corrected by M.A.J., O.V.G. and J.V.G. All authors have approved the final version of the manuscript and agree to be accountable for all aspects of the work in ensuring that questions related to the accuracy or integrity of any part of the work are appropriately investigated and resolved. All persons designated as authors qualify for authorship, and all those who qualify for authorship are listed.

### Funding

This work was supported by a Medical Research Council Programme Grant MR/J002771/1. O.H.P. is a Medical Research Council Professor (G19/22/2).

**Translational perspective**

Excessive Ca^2+^ signals and necrosis are the hallmark of acute biliary pancreatitis. Until now the adverse effects of the bile were attributed only to actions on pancreatic acinar cells and, to a lesser extent, duct cells. Indeed, necrotic death of acinar cells is particularly threatening for the pancreas, as it is associated with the release of activated digestive enzymes leading to extensive inflammation and autodigestion of the organ. Our data demonstrate for the first time that bile acids cause pathological Ca^2+^ signals and necrosis in pancreatic stellate cells, largely overlooked until recently. In fact, in this study stellate cells showed higher sensitivity, than adjacent acinar cells, to Ca^2+^ signals and necrotic death induced by the most abundant components of the bile: sodium cholate and taurocholate. Furthermore, triggering Ca^2+^ signals by bradykinin or induction of necrosis by sodium cholate in stellate cells was associated with exacerbated acinar cell death induced by taurolithocholic acid 3‐sulfate. Cholate‐ and taurocholate‐induced Ca^2+^ signals, as well as necrosis in stellate cells, were dependent on Na^+^‐driven bile uptake, most likely mediated by sodium–taurocholate cotransporting polypeptide (NTCP). Therapeutic intervention by temporary inhibition of pancreatic NTCP could therefore protect stellate cells from bile‐mediated killing and, at the same time, ameliorate necrosis in adjacent acinar cells. Our results also suggest that a substantial reduction in the severity of the early stages of acute biliary pancreatitis may be achieved by inhibition of pro‐inflammatory stimuli (such as bradykinin) that trigger Ca^2+^ signals in stellate cells.
